# Hospital Resources in the Eastern War

**Published:** 1912-10-19

**Authors:** 


### The Hospital

The Workers' Newspaper of Administrative Medicine and Institutional Life, Administration, National Insurance and Health.

No. 1372, Vol. LIII. SATURDAY, OCTOBER 19, 1912. PRICE ONE PENNY.

### HOSPITAL RESOURCES IN THE EASTERN WAR.

The outbreak of war in the Near East has directed attention to the resources of the conflicting Powers in connection with the care and treatment of the wounded. Since the Boer War our conception of the difficulties which the commissariat and Red Cross departments of an army may experience while within the zone of actual fighting has been considerably modified. Some of the lessons then learnt have been turned to good account in the Russian-Japanese and more lately in the Turkish-Italian Wars, but it seems probable that the new arrangements made as the result of the experience then gained will be severely tested during the conflict which is now in progress. With the exception of Turkey, whose Crescent service (since the red cross is displaced by the red half-moon in the Mohammedan department) is admirably equipped, staffed, and controlled, none of the combatants can claim to have developed what Baron Larrey called "the saving arm of the army" to the extent demanded by modern medical science. Roumania, which rumour states has thrown in her lot with Turkey, indeed rivals her ally in the completeness of her army ambulance service, and far surpasses that ally, or any of her possible antagonists, in the efficiency and training of her army surgeons. Bulgaria hardly possesses adequately staffed and equipped ambulance units which are equal to cope with the relatively large number of wounded that may be expected to demand treatment after an engagement. That this number will be proportionately greater than was the case during the Boer War is highly probable.

The nature of the fighting and the fact that large army corps will be engaged render it probable that the percentage of wounded will at least equal the figures that obtained during the Russian-Japanese War, when, as is a well-known fact, the fine field hospitals of the Russian army were unable to cope with the mass of work. Servia is slightly better off; her army nursing service is well trained and her ambulance units are thoroughly equipped—at least on paper—but she has too few units to be regarded as adequately provided for. Montenegro is much in the same condition as Bulgaria; her ambulances are sufficient for the standing troops but inadequate for the large number of reserves that will be called up. Greece, which has been making heroic efforts to be fit "to the last button" for war, has lately increased her ambulance department, and still more recently has had the invaluable help of private munificence and volunteer service. Through the interested activity of many patriotic men and women she has added to the number of her ambulances and provided hospital trains, emergency and base hospitals, and flying ambulance columns. Yet in her case, as in that of her possible allies, much remains to be done before her army can be said to be properly ambulanced. The necessity for extraneous aid and foreign charitable effort in the only line in which outsiders are privileged to take part in the struggle—the humanitarian part of caring for the wounded and sick and alleviating distress and suffering—is therefore apparent. Already the British Red Cross, with the approval of Queen Alexandra, has taken the initiative in offering its services to both sides, and there is little question that American and Continental societies will follow this generous and charitable example.

With the cessation of hostilities between Italy and Turkey, a consummation which is on the eve of realisation to judge by the latest reports, a number of voluntary ambulances now working in Tripoli will be released for service elsewhere and their staffs will no doubt be available for emergency work in Macedonia or wherever they may be most needed. Individual effort and private philanthropy will be able to render excellent service in the cause of humanity, and the British Red Cross deserves cordial support, both moral and financial, in the policy it has adopted by tendering its services. We have no doubt but that its offer will be accepted by one or other of the combatants who cannot afford to disregard or reject aid so precious and assistance so welcome in a department in which their own resources are obviously inadequate. The equipping and organisation of whatever voluntary ambulances are sent out need careful consideration. One of the great mistakes made is usually to sacrifice efficiency to economy or urgent convenience, but there can be no excuse for sending as a voluntary

Red Cross detachment a scratch ambulance team with faulty vehicles and bad outfit. One further point we think it necessary to urge and that is the desirability that volunteer ambulances should be provided with large and suitable Red Cross detachment flags. Experience in former wars has shown that the ordinary brassards are utterly inadequate to protect those serving under the Red Cross. They soon get dirty and indistinguishable, with the result that they are not seen in action and their wearers are fired upon. Unless this point is carefully attended to, we are convinced that there will in this war be no lack of the complaints of firing on Red Cross wagons, trains, and detachments, which have been a feature in every campaign since the Red Cross ensign flew on a battlefield. Such complaints are nearly always well founded, but the fault lies not with those who fire, but with those who, through neglect and thoughtlessness, allow the identity of the Red Cross sections to become obscured.

